# Supplementary material for: Genome-Scale Metabolic Modelling of Lifestyle Changes in Rhizobium leguminosarum
Source: mSystems. 2022 Jan 11;7(1):e00975-21. doi: 10.1128/msystems.00975-21 (PMC8751395; doi:10.1128/msystems.00975-21)
Supplement: TABLE S1 [file msystems.00975-21-st001.docx]

Table S1. Biomass composition for free-living *Rhizobium leguminosarum* bv. *viciae* 3841

| **Biomass component** | **Cellular content (g/g)** | **Reference** |
| --- | --- | --- |
| DNA | 0.028 | G. C. diCenzo, A. B. Benedict, M. Fondi, G. C. Walker, T. M. Finan, A. Mengoni, and J. S. Griffitts, PLOS Genet 14:e1007357, 2018, <https://doi.org/10.1371/journal.pgen.1007357> |
| RNA | 0.074 | K. Valgepea, K. Adamberg, A. Seiman, and R. Vilu, Mol Biosyst 9:2344–2358, 2013, <https://doi.org/10.1039/C3MB70119K> |
| protein | 0.55 | BNID 106154; R. Milo, P. Jorgensen, U. Moran, G. Weber, and M. Springer, Nucleic Acids Res 38:D750-D753, 2010, <https://doi.org/10.1093/nar/gkp889> |
| phospholipids | 0.091 | BNID 101436; R. Milo, P. Jorgensen, U. Moran, G. Weber, and M. Springer, Nucleic Acids Res 38:D750-D753, 2010, <https://doi.org/10.1093/nar/gkp889> |
| peptidoglycan | 0.025 | BNID 106154; R. Milo, P. Jorgensen, U. Moran, G. Weber, and M. Springer, Nucleic Acids Res 38:D750-D753, 2010, <https://doi.org/10.1093/nar/gkp889> |
| LPS | 0.015 | L. S. Forsberg and R. W. Carlson, J Biol Chem 283:16037–16050, 2008, <https://doi.org/10.1074/jbc.M709615200> |
| EPS | 0.05 | G. C. diCenzo, A. B. Benedict, M. Fondi, G. C. Walker, T. M. Finan, A. Mengoni, and J. S. Griffitts, PLOS Genet 14:e1007357, 2018, <https://doi.org/10.1371/journal.pgen.1007357> |
| beta-glucan | 0.0927 | M. W. Breedveld and K. J. Miller, Microbiol Rev 58:145–161, 1994, <https://doi.org/10.1128/mr.58.2.145-161.1994> |
| glycogen | 0.0386 | E. M. Lodwig, PhD thesis, University of Reading, 2001. |
| polyhydroxybutyrate | 0.0358 | E. M. Lodwig, PhD thesis, University of Reading, 2001. |
| Cofactors  NAD  NADP  FAD  S-adenosyl-methionine  CoA  FMN  riboflavin  tetrahydrofolate  10-formyl-tetrahydrofolate  5-methyl-tetrahydrofolate  thiamine diphosphate  pyridoxal-5-phosphate  hemeO  ubiquinone-8  biotinyl-5-AMP  lipoyl-acyl  carrier protein  cobalamin  UDP-N-acetylglucosamine  putrescine  phytoene  folate  homospermidine  putrescine  trace metals | Trace (1e^-5^) | J. C. Xavier, K. R. Patil, and I. Rocha, Metab Eng 39:200-208, 2017, <https://doi.org/10.1016/j.ymben.2016.12.002> |
