# Supplementary material for: Genome-Scale Metabolic Modelling of Lifestyle Changes in Rhizobium leguminosarum
Source: mSystems. 2022 Jan 11;7(1):e00975-21. doi: 10.1128/msystems.00975-21 (PMC8751395; doi:10.1128/msystems.00975-21)
Supplement: TABLE S2 [file msystems.00975-21-st002.docx]

Table S2. Compounds included in the *in silico* rhizosphere representation

| **Compound** | **ModelSEED ID** |
| --- | --- |
| shikimate | cpd00383 |
| protocatechuate | cpd00197 |
| mannitol | cpd00314 |
| arabinan | cpd12115 |
| tartrate | cpd00666 |
| homoserine | cpd00227 |
| tryptophan | cpd00065 |
| phenylalanine | cpd00066 |
| *myo*-inositol | cpd00121 |
| malonate | cpd00308 |
| fructose | cpd00082 |
| succinate | cpd00036 |
| malate | cpd00130 |
| proline | cpd00129 |
| alanine | cpd00035 |
| arginine | cpd00051 |
| aspartate | cpd00041 |
| asparagine | cpd00132 |
| cysteine | cpd00084 |
| glutamate | cpd00023 |
| glutamine | cpd00053 |
| glycine | cpd00033 |
| histidine | cpd00119 |
| isoleucine | cpd00322 |
| leucine | cpd00107 |
| lysine | cpd00039 |
| serine | cpd00054 |
| threonine | cpd00161 |
| valine | cpd00156 |
| glucose | cpd00027 |
| maltose | cpd00179 |
| raffinose | cpd00382 |
| sucrose | cpd00076 |
| xylose | cpd00154 |
| citrate | cpd00137 |
| fumarate | cpd00106 |
| glycolate | cpd00139 |
| arabinose | cpd00185 |
| fucose | cpd00751 |
| galactose | cpd00108 |
| mannose | cpd00138 |
| rhamnose | cpd00396 |
| *trans*-4-hydroxyproline | cpd00851 |
| tyrosine | cpd00069 |
| 5-oxoproline | cpd01293 |
| erythritol | cpd00392 |
| arabitol | cpd01307 |
| arabonate | cpd00651 |
| ribitol | cpd00366 |
| 2'-deoxyguanosine | cpd00277 |
| 2'-deoxyadenosine | cpd00438 |
| adenosine | cpd00182 |
| allantoin | cpd01092 |
| sorbitol | cpd00588 |
| xylitol | cpd00306 |
| lactate | cpd00159 |
| beta-hydroxypyruvate | cpd00145 |
| glycerate | cpd00223 |
| pantothenate | cpd00644 |
| betaine | cpd00540 |
| cytidine | cpd00367 |
| thymidine | cpd00184 |
| guanine | cpd00207 |
| urocanate | cpd00581 |
| allantoic acid | cpd00388 |
| 2'-deoxycytidine | cpd00654 |
| maltotriose | cpd01262 |
| uridine | cpd00249 |
| xanthosine 5'-monophosphate | cpd00497 |
| cytosine | cpd00307 |
| ribulose | cpd00258 |
| ribose | cpd00105 |
| orotate | cpd00247 |
| galactitol | cpd01171 |
| heme | cpd00028 |
| thymine | cpd00151 |
| glycerol | cpd00100 |
| 2'-deoxycytidine 5'-monophosphate | cpd00206 |
| gamma-aminobutyrate | cpd00281 |
| acetylphosphate | cpd00196 |
| pyridoxine | cpd00263 |
| cytidine 5'-monophosphate | cpd00046 |
| thymidine 5'-monophosphate | cpd00298 |
| 3-hydroxybutyryl CoA | cpd00842 |
| 3-hydroxybutyrate | cpd00797 |
| *cis*-vaccenate | cpd25615 |
| uracil | cpd00092 |
| inosine | cpd00246 |
| guanosine 5'- monophosphate | cpd00126 |
| ornithine | cpd00064 |
| thiamin diphosphate | cpd00056 |
| 2'-deoxyguanosine 5'-monophosphate | cpd00296 |
| methionine | cpd00060 |
| nicotinate | cpd00218 |
| xanthine | cpd00309 |
| O-acetylserine | cpd00722 |
| pyrophosphate | cpd00012 |
| 4-hydroxyphenylpyruvate | cpd00868 |
| anthranilate | cpd00093 |
| xanthosine | cpd01217 |
| 3-ureidopropionate | cpd01720 |
| diaminopimelate | cpd00504 |
| adenine | cpd00128 |
| 2-isopropylmalate | cpd01646 |
| argininosuccinate | cpd02152 |
| trehalose | cpd00794 |
| phosphopantetheine | cpd00834 |
| ethanolamine | cpd00162 |
| 5'-uridine monophosphate | cpd00091 |
| guanosine | cpd00311 |
| urate | cpd00300 |
| 2'-deoxyuridine | cpd00412 |
| 2'-deoxyinosine | cpd03279 |
| riboflavin | cpd00220 |
| putrescine | cpd00118 |
| agmatine | cpd00152 |
| adenosine 5'-monophosphate | cpd00018 |
| hypoxanthine | cpd00226 |
| Ca^2+^ | cpd00063 |
| Cl^-^ | cpd00099 |
| K^+^ | cpd00205 |
| Mg^2+^ | cpd00254 |
| Fe^2+^ | cpd10515 |
| SO_4_^2-^ | cpd00048 |
| Mn^2+^ | cpd00030 |
| Zn^2+^ | cpd00034 |
| Cu^2+^ | cpd00058 |
| MoO_4_ | cpd11574 |
| Co^2+^ | cpd00149 |
| Na^+^ | cpd00971 |
| biotin | cpd00104 |
| H^+^ | cpd00067 |
| PO_4_^3-^ | cpd00009 |
| O_2_ | cpd00007 |
