# Supplementary material for: Genome-Scale Metabolic Modelling of Lifestyle Changes in Rhizobium leguminosarum
Source: mSystems. 2022 Jan 11;7(1):e00975-21. doi: 10.1128/msystems.00975-21 (PMC8751395; doi:10.1128/msystems.00975-21)
Supplement: TABLE S3 [file msystems.00975-21-st003.docx]

Table S3. Compounds included in the *in silico* environment for nodule bacteria*^a^*

| **Compound** | **SEED ID** |
| --- | --- |
| xylose | cpd00154 |
| fructose | cpd00082 |
| sucrose | cpd00076 |
| erythritol | cpd00392 |
| mannitol | cpd00314 |
| *myo*-inositol | cpd00121 |
| formate | cpd00034 |
| malonate | cpd00308 |
| malate | cpd00130 |
| succinate | cpd00036 |
| fumarate | cpd00106 |
| tartrate | cpd00666 |
| GABA | cpd00281 |
| phenylalanine | cpd00066 |
| tyrosine | cpd00069 |
| tryptophan | cpd00065 |
| leucine | cpd00107 |
| valine | cpd00156 |
| isoleucine | cpd00322 |
| alanine | cpd00035 |
| aspartate | cpd00041 |
| glycine | cpd00033 |
| glutamate | cpd00023 |
| glutamine | cpd00053 |
| asparagine | cpd00132 |
| histidine | cpd00119 |
| proline | cpd00129 |
| serine | cpd00054 |
| threonine | cpd00161 |
| methionine | cpd00060 |
| cysteine | cpd00084 |
| lysine | cpd00039 |
| arginine | cpd00051 |
| xanthosine | cpd01217 |
| 2-isopropylmalate | cpd01646 |
| uridine | cpd00249 |
| uracil | cpd00092 |
| agmatine | cpd00152 |
| hypoxanthine | cpd00226 |
| adenine | cpd00128 |
| homoserine | cpd00227 |
| H^+^ | cpd00067 |
| heme | cpd00028 |
| inosine | cpd00246 |
| argininosuccinate | cpd02152 |
| guanosine | cpd00311 |
| O-acetylserine | cpd00722 |
| ethanolamine | cpd00162 |
| thiamin diphosphate | cpd00056 |
| pyridoxine | cpd00263 |
| homocitrate | cpd00919 |
| biotin | cpd00104 |
| pantothenate | cpd00644 |
| phosphopantetheine | cpd00834 |
| niacin | cpd00218 |
| riboflavin | cpd00220 |
| O_2_ | cpd00007 |
| PO_4_^3-^ | cpd00009 |
| Cu^2+^ | cpd00058 |
| Ca^2+^ | cpd00063 |
| Cl^-^ | cpd00099 |
| Co^2+^ | cpd00149 |
| K^+^ | cpd00205 |
| Mg^2+^ | cpd00254 |
| N_2_ | cpd00528 |
| Na^+^ | cpd00971 |
| Fe^2+^ | cpd10515 |
| MoO_4_ | cpd11574 |
| Zn^2+^ | cpd00034 |
| SO_4_^2-^ | cpd00048 |

*^a^*The list of compounds is based on the induction of rhizobial biosensors in pea nodules and metabolomics data for the nodule cytosol. In addition, all amino acids and vitamins present in the nodule cytosol were included.
