# Supplementary material for: Genome-Scale Metabolic Modelling of Lifestyle Changes in Rhizobium leguminosarum
Source: mSystems. 2022 Jan 11;7(1):e00975-21. doi: 10.1128/msystems.00975-21 (PMC8751395; doi:10.1128/msystems.00975-21)
Supplement: TABLE S4 [file msystems.00975-21-st004.docx]

Table S4. Nutrients available to bacteroids*^a^*

| **Compound** | **SEED ID** |
| --- | --- |
| xylose | cpd00154 |
| erythritol | cpd00392 |
| mannitol | cpd00314 |
| *myo*-inositol | cpd00121 |
| formate | cpd00034 |
| malonate | cpd00308 |
| malate | cpd00130 |
| succinate | cpd00036 |
| fumarate | cpd00106 |
| tartrate | cpd00666 |
| GABA | cpd00281 |
| phenylalanine | cpd00066 |
| tyrosine | cpd00069 |
| tryptophan | cpd00065 |
| leucine | cpd00107 |
| valine | cpd00156 |
| isoleucine | cpd00322 |
| alanine | cpd00035 |
| aspartate | cpd00041 |
| glycine | cpd00033 |
| glutamate | cpd00023 |
| glutamine | cpd00053 |
| asparagine | cpd00132 |
| histidine | cpd00119 |
| proline | cpd00129 |
| serine | cpd00054 |
| threonine | cpd00161 |
| methionine | cpd00060 |
| cysteine | cpd00084 |
| lysine | cpd00039 |
| arginine | cpd00051 |
| glycolate | cpd00139 |
| pantothenate | cpd00644 |
| phosphopantetheine | cpd00834 |
| H^+^ | cpd00067 |
| heme | cpd00028 |
| Mn^2+^ | cpd00030 |
| O_2_ | cpd00007 |
| PO_4_^3-^ | cpd00009 |
| thiamin diphosphate | cpd00056 |
| Cu^2+^ | cpd00058 |
| Ca^2+^ | cpd00063 |
| Cl^-^ | cpd00099 |
| biotin | cpd00104 |
| Co^2+^ | cpd00149 |
| K^+^ | cpd00205 |
| niacin | cpd00218 |
| riboflavin | cpd00220 |
| Mg^2+^ | cpd00254 |
| pyridoxine | cpd00263 |
| N_2_ | cpd00528 |
| homocitrate | cpd00919 |
| Na^+^ | cpd00971 |
| Fe^2+^ | cpd10515 |
| MoO_4_ | cpd11574 |
| Zn^2+^ | cpd00034 |
| SO_4_^2-^ | cpd00048 |

*^a^*The list of compounds is based on the induction of rhizobial biosensors in nodules. In addition, all amino acids and vitamins present in the nodule cytosol were included.
