# Supplementary material for: Genome-Scale Metabolic Modelling of Lifestyle Changes in Rhizobium leguminosarum
Source: mSystems. 2022 Jan 11;7(1):e00975-21. doi: 10.1128/msystems.00975-21 (PMC8751395; doi:10.1128/msystems.00975-21)
Supplement: TABLE S5 [file msystems.00975-21-st005.docx]

Table S5. Enrichment of amino acids in Nif and Fix proteins*^a^*

| **Amino acid** | **Overall percentage in coding sequences [%]** | **Percentage in Nif and Fix proteins [%]** | **Adjusted *P* value** |
| --- | --- | --- | --- |
| alanine | 11.81 | 10.22 | 0.9998 |
| cysteine | 0.82 | 1.53 | 3.0026*10^-6^ |
| aspartate | 5.70 | 6.08 | 0.3860 |
| glutamate | 5.72 | 5.94 | 0.5768 |
| phenylalanine | 3.94 | 3.31 | 0.9998 |
| glycine | 8.24 | 8.75 | 0.3460 |
| histidine | 2.04 | 2.57 | 0.0420 |
| isoleucine | 5.79 | 5.96 | 0.6195 |
| lysine | 3.68 | 5.14 | 2.4365*10^-6^ |
| leucine | 9.95 | 9.16 | 0.9998 |
| methionine | 2.57 | 2.77 | 0.4936 |
| asparagine | 2.85 | 3.39 | 0.0681 |
| proline | 4.90 | 4.44 | 0.9998 |
| glutamine | 3.09 | 3.15 | 0.7740 |
| arginine | 6.77 | 6.06 | 0.9998 |
| serine | 5.89 | 5.62 | 0.9998 |
| threonine | 5.33 | 5.20 | 0.9998 |
| valine | 7.24 | 6.89 | 0.9998 |
| tryptophan | 1.32 | 1.22 | 0.9998 |
| tyrosine | 2.33 | 2.63 | 0.3460 |

*^a^*The amino acid compositions of all coding sequences in the Rlv3841 genome and the Nif and Fix proteins were compared using a hypergeometric test followed by Benjamini-Hochberg false discovery rate correction.
